# Supplementary material for: SyNDI: synchronous network data integration framework
Source: BMC Bioinformatics. 2018 Nov 6;19:403. doi: 10.1186/s12859-018-2426-5 (PMC6219086; doi:10.1186/s12859-018-2426-5)
Supplement: Supplementary file 5 — Files of the example presented in Additional file 4. This additional file is a zip package that contains all files used in Additional file 4. Detailed descriptions of each of these files are presented in a README file included in this zip package. (GZ 30411 kb) [file 12859_2018_2426_MOESM5_ESM.gz › SyNDITools/Meme2Fimo/meme_4.9.0_4.tar.gz!/meme_4.9.0/doc/compute-prior-dist.xml]

compute-prior-dist


# compute-prior-dist

**Usage:** `compute-prior-dist <numbins> <psp-filename>`

**Description:**

`compute-prior-dist` computes the distribution of priors from a file in
MEME PSP format.
The disbution is scaled and binned into the number of bins specified on the command line.

**Input:**

The PSP file must be in MEME PSP format.

**Output:**

The output is sent to `stdout`,
and contains one `double` per line.
The first two lines are the minimum and maximum priors.
Each succeeding line contains the probability that a prior
is in the bin.
The prior is derived from the bin number using the formula

```
prior = ((bin #) / (num bins - 1) * (max prior - min prior)) + (min prior)
```

(bins are numbered staring at 0).

Sample output
